# Phytochemical Profile, Antiradical Capacity and α-Glucosidase Inhibitory Potential of Wild *Arbutus unedo* L. Fruits from Central Italy: A Chemometric Approach

**DOI:** 10.3390/plants9121785

**Published:** 2020-12-16

**Authors:** Valentina Macchioni, Veronica Santarelli, Katya Carbone

**Affiliations:** 1 CREA Research Centre for Olive, Fruit and Citrus Crops, Via di Fioranello 52, 00134 Rome, Italy; valentina.macchioni@crea.gov.it; 2 Faculty of Bioscience and Technologies for Food, Agriculture and Environment, University of Teramo, Via Renato Balzarini 1, 64100 Teramo, Italy; vsantarelli@unite.it

**Keywords:** *A. unedo*, strawberry tree fruit, polyphenols, nutraceuticals, wild berries, antiradical capacity, a-glucosidase inhibition

## Abstract

Nowadays, there is a growing interest in botanicals for human nutrition and care. *Arbutus unedo* wild berries are edible and medicinal fruits that contain many healthy bioactive components, which can be considered a valuable resource for the food ingredient market and for nutraceutical and cosmetic sectors. In the present study, the polyphenols and in vitro antiradical and hypoglycemic activities of five wild Italian accessions of *A. unedo* were investigated, and their chemical profiles were treated by means of unsupervised chemometric techniques like the hierarchical and principal component analysis. Moreover, Fourier-transformed mid-infrared spectroscopy was used to provide a rapid assessment of the phytochemical composition of different accessions. Samples differed mainly in their anthocyanin content and overall nutraceutical potential. Anthocyanins were present mainly as glycosides of cyanidin and delphinidin, with delphinidin-3-O-glucoside being the most abundant one, ranging from 49 ± 1 to 111 ± 3 mg g^−1^ (for P1 and P2, respectively; *p* < 0.05). Extracts were screened for their in vitro biological activities by using the 2,2′-azino-bis (3-ethylbenzothiazoline-6-sulfonic acid) (ABTS^•+^), 2,2-diphenyl-1-picrylhydrazyl (DPPH^•^) antiradical tests, while their hypoglycemic activity was investigated by the α-glucosidase inhibition test. In both in vitro antiradical tests, the highest capacity was recorded for P2 (EC_50_: 1.17 and 0.064 mg mL^−1^, for DPPH^•^ and ABTS^•+^, respectively), with values higher than those reported in the literature for *A. unedo* fruit extracts. P2 also showed the highest inhibition power towards α-glucosidase (about 70%). Moreover, the nonparametric correlation analysis pointed out a very high significant correlation between the percentage of α-glucosidase inhibition and cyanidin-3-O-rutinoside (r: 0.973; *p* < 0.01). Finally, the application of hierarchical analysis to samples analyzed provided three different clusters based on the average phytochemical content coded as low, medium and high. Moreover, principal component analysis made it possible to establish similarities among the accessions depending on their overall nutraceutical characteristics and on the relative anthocyanin content.

## 1. Introduction

*Arbutus unedo* L. (Ericaceae family), commonly known as the strawberry tree, is a wild evergreen shrub native to the Mediterranean region, which grows spontaneously in other regions characterized by hot summers and mild and rainy winters [1]. It is a xerophilous plant that prefers siliceous soils and grows at altitudes between 0 and 800 m. In Italy, its diffusion is continuous along the Ligurian, Sardinian, Sicilian, Tyrrhenian and Adriatic coasts. From an ecological point of view, *A. unedo* L. is a hardy plant, and this plays a key role in reforestation programs in southern European countries such as Greece, Italy, Portugal and Spain, where forest fires are common during the dry season [2]. *A. unedo* L. produces red spherical fruits, having a diameter of about 2–3 cm. These fruits are rarely eaten fresh, despite their pleasant taste when fully ripe, because they are rich in seeds and therefore not very appreciated by the modern consumer [3]. However, they have some importance in local agricultural communities, which use them for the production of alcoholic beverages, jams, jellies and marmalades [4]. Moreover, these fruits have been known since ancient times for their healthy properties, being used in folk medicine for diverse purposes [5] and the Food and Agriculture Organization (FAO) is currently looking for ways to increase the use of this precious wild species [6], with the aim to preserve plant biodiversity.

Recent studies suggest that strawberry tree’s bioactive compounds are linked to a broad spectrum of human health benefits such as antidiabetic, antihypertensive, anti-inflammatory, antitumor, antioxidant and spasmolytic [5], antiseptic, diuretic and laxative properties [7]. The potential advantages of *A. unedo* fruits are related to the presence of polyphenolic compounds in its composition [5], capable of eliminating oxygen radicals and other reactive species. The main phenolic compounds in strawberry tree fruits are anthocyanins [8]. These bioactive substances are proved to be inhibitors of α-glucosidase, contributing to the reduction of type-2 diabetes [9,10]. Moreover, the European Union allows the use of anthocyanins as food colorants (namely E163) in acidic food or beverages, including soft drinks, fruit preserves, sugar confectioneries, dairy products, frozen products, dry mixes and alcoholic drinks. Anthocyanins have not only shown beneficial effects as antioxidants [11] but also, they have the ability to regulate the gene expression of adipocytokines [12]. Hence, there is little to no data in literature on the antioxidants present in the fruit of *A. unedo* L., especially those of spontaneous Italian flora, although its antioxidant content is very high compared to other fruits [13]. The formulation of phytoextracts rich in bioactive compounds is of fundamental importance to promote the use of these underutilized fruits in various industrial sectors, being a precious resource for the food ingredient market and for nutraceutical and cosmetic sectors. To the best of our knowledge, there is very limited information on the antiradical potential and nutraceutical attributes of wild *A. unedo* fruits growing in Italy [14] and on the relationships among these attributes and the biological activity linked to them, whereas the search for new resilient crops able to adapt to the strong climate change is increasingly pressing. In light of these considerations, the present study aimed to evaluate the content of phenols and anthocyanins in fruit extracts from five spontaneous accessions of *Arbutus unedo* L., growing in Central Italy, through a comparative analysis of their phenolic profile, antiradical potential and hypoglycemic activity. Finally, the relationships among analyzed parameters were carried out by using a chemometric approach based on hierarchical cluster analysis (HCA) and principal component analysis (PCA).

## 2. Materials and Methods

### 2.1. Chemicals

Folin–Ciocâlteu reagent, gallic acid, catechin, 2,2-diphenyl-1-picrylhydrazyl radical (DPPH^•^), vanillin, 2,2′-azinobis-(3-ethylbenzothiazolin-6-sulfonic acid) (ABTS^•+^), potassium persulfate, and vanillin were purchased from Sigma-Aldrich (Milan, Italy). Delphinidin-3-O-glucoside chloride, cyanidin 3-O-glucoside chloride, cyanidin-3-O-rutinoside chloride and malvidin-3-O-glucoside chloride standards used for identification and quantification purposes with high-performance liquid chromatography (HPLC) were purchased from Extrasynthese (Genay, France). Organic solvents used for chromatography were of HPLC ultragradient grade (Sigma-Aldrich, Milan, Italy), whereas distilled water was obtained by the Milli-Q system (Millipore, Milan, Italy). The 45 µm pore size membrane filters were purchased from Pall (Pall Corporation, Ann Arbor, MI, USA) and used for filtration of samples.

### 2.2. Plant Material

In the present study, fresh and healthy fruits of *A. unedo*, fully ripened (average total soluble solids: 17.1 ± 0.7 °Brix; skin fruit’s color: homogenous red-scarlet pigmentation), were collected randomly from plants (five accessions, indicated with the letter “P”) growing wild along the Lazio coast (Central Italy; 41°52’47“N 12°16’29” E; 70 m a.s.l.) and taxonomically identified. Fruits were kept in a cooler bag during the transport to the laboratory. Four lots of 30 fruits per accession were prepared and sorted based on homogeneous size and absence of physical injuries and/or infections. Soon after harvested, samples were frozen in liquid nitrogen, freeze-dried and ground prior to analysis.

### 2.3. Fourier–Transform Infrared Spectroscopy (FTIR) Analysis in Attenuated Total Reflectance (ATR) Mode

ATR-FTIR spectra of *A. unedo* wild berries were acquired as described by Amoriello et al. [15] without modifications. IR spectra (wavenumbers ranging from 4000 to 600 cm^−1^) were collected at room temperature with a FT-IR spectrometer (iS 50 FT-IR Nicolet Thermo Fisher Scientific Inc.,Milan, Italy) equipped with a single-reflection horizontal ATR cell with a diamond crystal. After the acquisition, processed spectral data were obtained with the OMNIC™ software (Thermo Fisher Scientific Inc., Milan, Italy).

### 2.4. Extraction of Bioactive Compounds

Freeze-dried samples were extracted to determine their phytochemical composition, e.g., total polyphenol content (TPC), total flavan-3-ol content (FLC), total monomeric anthocyanins (TMA), HPLC with diode-array detection polyphenol profile, antiradical capacity (AC) and α-glucosidase inhibitory activity (AGA). 1.0 g of fruit powder was extracted with 15 mL of a hydroalcoholic solution (methanol: water = 80:20, *v/v*) acidified with 0.1% HCl (*v/v*), for 30 min, under continuous mechanical stirring at room temperature (shaking incubator mod. SKI 4; Argolab, Milan, Italy), followed by 30 min of ultrasound-assisted extraction, performed in a temperature-controlled sonication bath (UTA-200, Falc, Italy), operating at 40 kHz. The resulting extracts were then centrifuged at 6792 *g* for 15 min at 4 °C. The extraction procedure was repeated twice using 15 mL of fresh solvent, and the two extracts were combined and filtered through Whatman No. 1 paper immediately prior to all analyses.

### 2.5. Total Phenolic Content

The total content of soluble polyphenols in the extracts was determined, according to Carbone et al. [16]. TPC was calculated using a calibration curve of gallic acid. The results were expressed as milligrams of gallic acid (GAE) equivalents per gram of fruit powder (mg GAE g^−1^). All determinations were performed in triplicate.

### 2.6. Total Flavan-3-ol Content

FLC was determined following the vanillin assay method, as reported by Carbone et al. [16]. FLC was calculated from a calibration curve, using catechin as a standard. Results were expressed as milligrams of catechin equivalents per gram of fruit powder (mg CTE g^−1^). All determinations were performed in triplicate.

### 2.7. Total Monomeric Anthocyanin Content

TMA content of the extracts was performed according to Ciccoritti et al. [10], without modifications. Data were expressed as μg cyanidin-3-O-glucoside (cyd-3-glu) equivalents g^−1^ of fruit powder. All determinations were performed in triplicate.

### 2.8. HPLC-DAD Profiles of Single Phenols and Anthocyanins

Anthocyanins were separated and identified by an analytical HPLC system (Agilent 1100 series, Agilent, Milan, Italy) equipped with a diode array detector (DAD) (Agilent Technologies, Milan, Italy). The separation was carried out on a Zorbax SB C18 column (Agilent, 4.6 × 250 mm; 5 μm particle size, set at 30 °C), according to Ciccoritti et al. [10]. Single anthocyanins were identified at 520 nm by their retention times and spectral data as compared to individual standards and by the method of standard additions to the samples.

The single phenols of the different extracts were separated and identified by an HPLC analytical system (Agilent 1100 series, Agilent, Italy) equipped with a DAD (Agilent Technologies, Italy), which was fixed simultaneously at 280 nm (benzoic acids and flavan-3-ols), 320 nm (hydroxycinnamic acids) and 370 nm (flavonols). In addition, UV-vis spectra were recorded in the range of 200–700 nm. The separation of single phenols was performed according to the protocol reported by Carbone and Mencarelli [17], without modifications, on a Luna C18 column (Phenomenex; 4.6 × 250 mm; particle size 5 µm, set at 30 °C). The injection volume was 20 μL.

All analytical data were evaluated using a chromatographic data management system (Chemstation 32.1, Agilent Technologies). Ten-point calibration curves based on external standard solutions (0–100 ppm) were obtained for quantification.

Results were expressed as mg g^−1^ (for phenolics) and as μg g^−1^ (for anthocyanins) of fruit powder.

### 2.9. In Vitro Bioactivity of A. unedo Fruit Extracts

#### 2.9.1. Antiradical Capacity (AC) Assays

The radical scavenging power of the samples was assessed by measuring their ability to scavenge synthetic radicals (e.g., DPPH^•^ and ABTS^•+^), according to Carbone et al. (2011) [16]. Results were expressed as EC_50_. All determinations were performed in triplicate.

#### 2.9.2. α-Glucosidase Inhibition Assay

The α-glucosidase activity of analyzed samples was evaluated according to the Sigma-Aldrich enzymatic assay for α-glucosidase [18], using *p*-nitrophenyl α-D-glucoside as substrate. The α-glucosidase activity was spectrophotometrically determined by measuring the release of *p*-nitrophenol from *p*-nitrophenyl α-D-glucopyranoside at 400 nm. Inhibition of the enzyme activity was expressed as percentage inhibition and calculated as follows:

α-glucosidase inhibition (%) = (Abs_control_ − Abs_sample_)/(Abs_control_) × 100.

All determinations were performed in triplicate.

### 2.10. Statistical Analysis

Statistical analysis was performed with SPSS 25.0 software (SPSS, Inc., Chicago, Illinois). Data were reported as means ± standard deviation (SD) of four independent experiments with three replicates. Prior to chemometric applications, all variables were auto-scaled (transformation into z-scores) to standardize the statistical importance of all responses. An exploratory data analysis was made to check the data-normal distribution (Kolmogorov–Smirnov test) and the homogeneity of variance (Levene’s test). Data violated ANOVA assumptions, even after their mathematical transformation. In light of the obtained results, we analyzed non-normally distributed data through the Kruskal–Wallis (K-W) nonparametric test, while significant mean differences were established using the Mann–Whitney test for independent and nonparametric procedures (*p* < 0.0167 for Bonferroni’s correction, where not differently specified; [19]). Correlations among all parameters in the dataset were analyzed using Spearman’s correlations (*r*; *p* < 0.05 and *p* < 0.01). Finally, principal component analysis (PCA) and hierarchical cluster analysis (HCA) were used as exploratory chemometric methods to study the data structure, investigating similarities and hidden patterns among the extracts analyzed. Both methods were applied to normalized data (z-scores) to retrieve all chemically relevant information systematically, using SPSS 25.0 software (SPSS, Inc., Chicago, IL). Clusters were computed by Ward’s method based on Euclidean distance, and the significant differences among clusters were investigated by K-W test followed by Dunn’s test for post-hoc analyses.

## 3. Results and Discussion

### 3.1. FTIR Spectra Analysis

**Figure 1:**
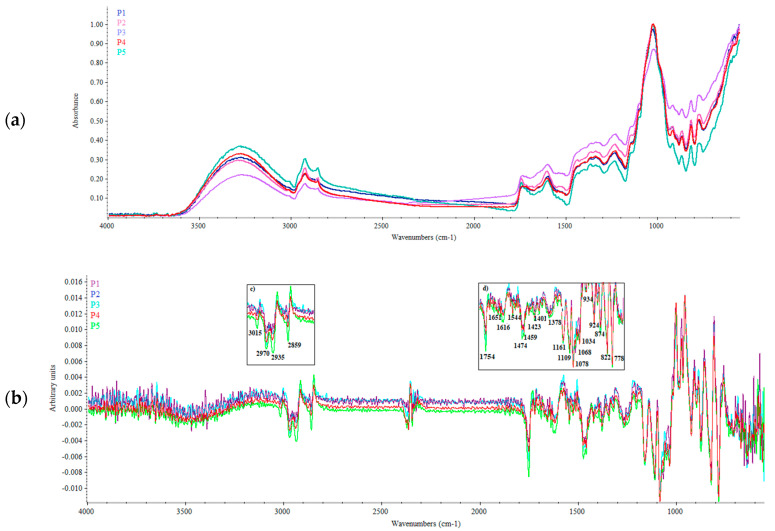
Average IR spectra of *A. unedo* wild berries analyzed: (**a**) raw spectra; (**b**) first derivative spectra.

In the present study, ATR-FTIR analysis of *A. unedo* wild berries was carried out, for the first time, to provide effective information about the qualitative composition of the plant matrix by analyzing the relation between the spectral fingerprints and molecular structures of the samples. Figure 1a,b shows the absorbance (a) and first derivative spectra (b) of samples analyzed, acquired in the mid-infrared region (see also Table S1 in the electronic-supplementary material). The first derivative IR spectra allowed the analysis of overlapped peaks. The spectra were analyzed with respect to the spectral band positions in order to identify the signatures of the major functional groups characterizing the *A. unedo* berries. As can be seen (Figure 1a), the unique spectral patterns of extracts from different wild accessions reflected their compositional differences (different band positions and intensities), suggesting significant genetic differences.

Two characteristic spectral regions are clearly evident above 2800 cm^−1^ and below 1800 cm^−1^ (Figure 1a); the latter indicative of the functional groups and fingerprinting of the plant matrix analyzed. The first broad band located at about 3300 cm^−1^ is attributed to the O-H stretching, which is associated with the presence of carboxyl groups in compounds such as polyphenol acids, as well as to the O-H stretching vibration of the phenolic groups, which are present in *A. unedo* wild berries [5]. A band system around 3000–2800 cm^−1^, with two weak and broad peaks centered at 2970 and 2935 cm^−1^ related to the symmetric and asymmetric stretching of -CH_3_ and -CH_2_- groups, characteristics of lipids and fatty acids, was also visible (Figure 1b; inset c). In this region, the first derivative spectra pointed out also a defined peak at 2859 cm^−1^, preceded by a shoulder centered at about 2870 cm^−1^, characterizing the presence of alkanes (C–H) and aldehydic groups (–CO–H), as reported by Umdale et al. [20]. Moreover, the derivative spectra pointed out the presence of a small but clear band to the left of the system of intense signals due to aliphatic stretching, i.e., just above 3000 cm^−1^ (3015 cm^−1^; Figure 1b, inset c), occurring by the presence of alkene terminal groups when a single H atom was bound to the double bond. These findings agree with those of Morgado et al. [5], who reported a high content of polyunsaturated fatty acids, representing about 60% of total fatty acids, in *A. unedo* berries.

The fingerprinting region between 1800 and 700 cm^−1^ showed significant differences between the samples analyzed (Figure 1b, inset d). A well-defined peak, occurring at 1750 cm^−1^, related to the carbonyl group (C=O) stretching vibrations of the ester bond, was present in all extracts analyzed. The presence of esters was also confirmed by the strong absorption at 1078 cm^−1^ (stretching of COO-C) (Figure 1b, inset d). These findings are in line with the fact that phenolics, especially phenolic acids, occur naturally in combination with other compounds, usually in the form of esters [21]. The low-intensity absorption bands in the range 1500–1450 cm^−1^ could be attributed to C–C-O stretching vibrations [22]. From derivative spectrum (Figure 1b; inset d), several peaks in the spectral range 1100–1000 cm^−1^, due to the stretching of OH group, characteristic of phenolics, and to the aromatic C–H-plane deformation vibrations, were also observed [23]. Finally, a well-defined band system in the range 900–750 cm^−1^ was due to the oscillations of the H atoms outside the aromatic ring plane, depending on the number of adjacent H atoms and as a consequence on the meta substitution of aromatic protons [21].

### 3.2. Phytochemical Composition of the Extracts Analyzed

**Figure 2:**
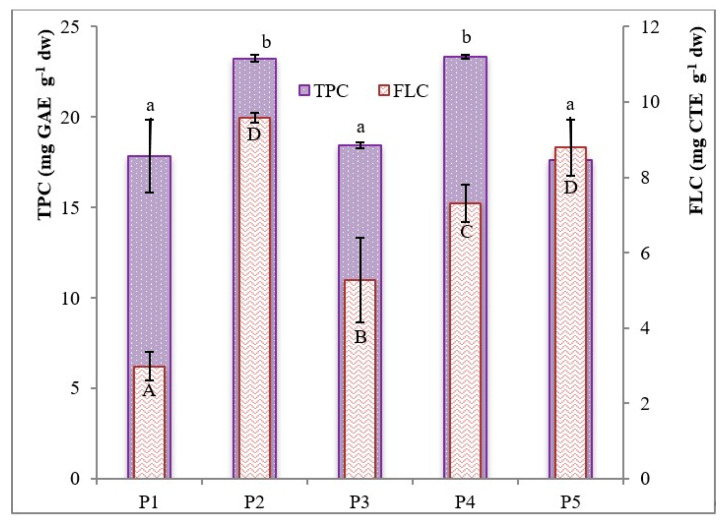
Total phenolic and flavan-3-ols content (mg g^−1^) of five *A. unedo* fruit extracts (mean ± SD). Different letters indicate significant differences in the mean (*p* < 0.05). TPC: total polyphenol content; FLC: flavan-3-ols content; GAE: gallic acid equivalents; CTE: catechin equivalents.

Figure 2 shows the TPC of the different extracts analyzed, which ranged from 17.6 ± 0.3 to 23.3 ± 0.2 mg GAE g^−1^ (for P5 and P4, respectively).

Based on the experimental results, the genotype significantly influenced the TPC of the extracts (*p* < 0.05) as follows: P4~P2 > P3 > P1 > P5. These results are in agreement with those reported in the literature [7] [24] and higher than those reported by Doukani et al. [25], which ranged between 7 and 14 mg GAE g^−1^. As regards flavan-3-ols, FLC ranged from 2.98 to 9.58 mg CTE g^−1^ (for P1 and P2, respectively; *p* < 0.05), being P2 > P5 > P4 > P3 > P1 (Figure 2). Reported values are higher than those found by Zitouni et al. [26] for wild Moroccan accessions. Moreover, it is interesting to note that the FLC of the P5 accession represented about 50% of its TPC. Such high values of flavans can be of considerable phytotherapeutic importance; in fact, it is known that these compounds are associated with a reduced risk of stroke, myocardial infarction and diabetes, as well as with improvements in lipid profiles, endothelium-dependent blood flow and blood pressure, insulin resistance and systemic inflammation.

**Figure 3:**
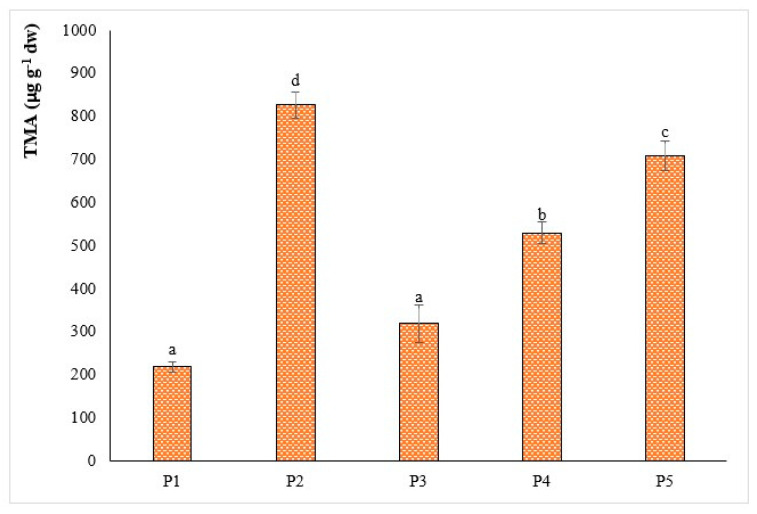
Total content (μg g^−1^) of monomeric anthocyanins of different *A. unedo* fruit extracts (mean ± SD). Different letters indicate significant differences in the mean (*p* < 0.05). TMA: total monomeric anthocyanin.

In the present study, the anthocyanin content of different extracts tested ranged between 219 ± 8 and 827 ± 3 μg g^−1^ fruit (for P1 and P2, respectively; Figure 3).

On average, the TMA of extracts analyzed was higher than those obtained by Lopez et al. [27] with optimized ultrasound extraction. Moreover, the anthocyanin content of the wild fruit analyzed was within the same range of known fruits characterized by a high anthocyanin content, such as *Nitraria tangutorum* Bobr. (~650 μg g^−1^ fruit dw) [28] and *Aristotelia chilensis* L. (400–1500 μg g^−1^ fruit dw) [29]. These findings are of particular interest due to the role of anthocyanins in the control of obesity, the control of diabetes, the prevention of cardiovascular diseases and the improvement of visual and brain functions.

Overall, the differences found in the phytochemical parameters analyzed could be due to differences in the genetic traits of the different *A. unedo* accessions analyzed.

### 3.3. Chromatographic Profiling of Single Phenols

**Figure 4:**
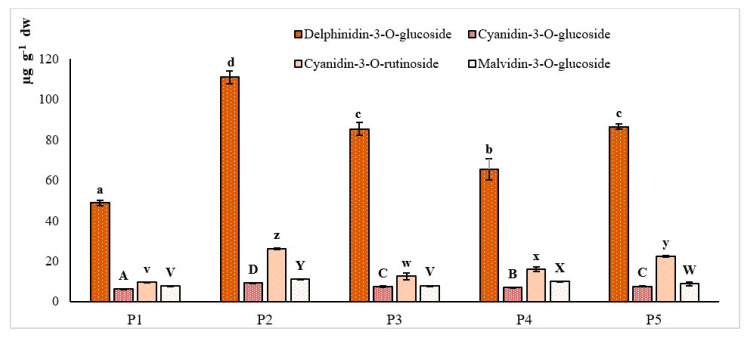
HPLC–DAD quantification of individual anthocyanins (μg g^−1^) of different extracts analyzed (mean ± SD).

**Figure 5:**
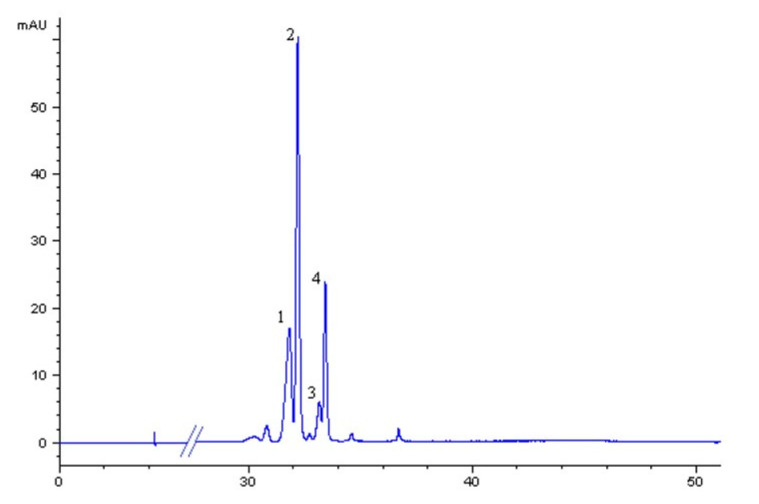
A typical HPLC-DAD chromatogram (λ: 520 nm) of the extracts analyzed. The operating conditions are reported in the HPLC section. Peak assignment: 1: delphinidin-3-O-glucoside, 2: cyanidin-3-O-glucoside; 3: cyanidin-3-O-rutinoside; 4: malvidin-3-O-glucoside.

As far as anthocyanins are concerned, *A. unedo* fruits are mainly characterized by the presence of cyanidin and delphinidin glycosides [30]. In the present study, the content and abundance of different anthocyanins in the wild *A. unedo* fruit extracts, including delphinidin-3-O-glucoside, cyanidin-3-O-glucoside, malvidin-3-O-glucoside and cyanidin-3-O-rutinoside, were determined by HPLC-DAD (Figure 4 and Figure 5).

The main anthocyanin found in all the extracts tested was delphinidin-3-O-glucoside, ranging from 49 ± 1 to 111 ± 3 µg g^-1^ (for P1 and P2, respectively; *p* < 0.05), followed by cyanidin-3-O-rutinoside, ranging from 9.5 ± 0.3 to 26.2 ± 0.4 µg g^−1^ (for P1 and P2, respectively; *p* < 0.05). These features are of particular interest as literature data report cyanidins as the main components of strawberry fruit extracts [30] [31], highlighting peculiar nutraceutical traits linked to genetic features of accessions studied. Due to their antioxidant potential, delphinidin glucosides showed the strongest scavenging activity against superoxide anion and peroxynitrite among several anthocyanins [32]. Moreover, delphinidin was reported to exert cytoprotective effects for human chondrocytes against oxidative stress [33], as well as inhibitory effects on oxidative stress in HepG2 cells [34]. To the best of our knowledge, this is the first study reporting malvidin-3-O-glucoside in *A. unedo* wild fruit extracts. On average, extracts analyzed were characterized by a malvidin-3-O-glucoside content of 9.1 ± 0.3 µg g^−1^, with P2 showing the highest value (11.1 ± 0.4 µg g^−1^). From a nutritional point of view, anthocyanins, as well as the other phenolic classes, are absorbed at the intestinal tract level, where they are hydrolyzed, and the aglycones (anthocyanidins) are subjected to phase II metabolism [35]. Among anthocyanidins, delphinidin and malvidin were found to exhibit the highest growth-inhibitory potential towards neoplastic cell survival [36].

According to literature studies, several classes of phenolic compounds were detected in strawberry tree extracts, including hydroxybenzoic acids (gallic, protocatechuic, syringic acids, hydroxybenzoic acids), hydroxycinnamic acids (chlorogenic, *p*-coumaric, ferulic and caffeic acids), flavanols (catechin and epigallocatechin) [37]. Significant variations in phenolic compounds were found at *p* < 0.001 among wild accessions analyzed. In detail, P2 showed the highest polyphenol content (4.46 mg g^−1^) and P1 the lowest one (2.16 mg g^−1^) (Table 1).

Among the phenolic classes analyzed, hydroxybenzoic acids (HBA) were the most abundant compounds, followed by flavan-3-ols and hydroxycinnamic acids (HCA), while no appreciable amounts of flavonols were found. These results are in agreement with literature data [38].

The highest HBA content was found in P4 (2.78 ± 0.06 mg g^−1^) and the lowest one in P1 (1.2 ± 0.1 mg g^−1^). Among the hydroxybenzoic acids, gallic acid was the most representative (ranging from 0.31 to 1.29 mg g^-1^, for P5 and P4, respectively) as also reported by Pimpão et al. [35]. Moreover, among HCA analyzed, only chlorogenic acid was found, but at much lower levels than those reported by Zitouni et al. [26] for wild Moroccan accessions.

As far as flavan-3-ols are concerned, catechin was the most abundant compound found in all the extracts analyzed, being also the most representative phenolic compound in wild accessions studied. Its content ranged from 0.82 to 1.72 mg g^−1^ for P1 and P2, respectively. This trend was similar to that previously observed for FLC, and obtained values were on average higher than those reported in literature studies [26] and in line with the values reported by Albuquerque et al. [39] under optimal extraction conditions. Flavonols are generally present in low amounts in *A. unedo* berries [26]. In the present study, myricetin was not found in any of the extracts tested, whereas other not identified flavonols were detected only in traces (data not shown).

### 3.4. In Vitro Biological Activities

**Figure 6:**
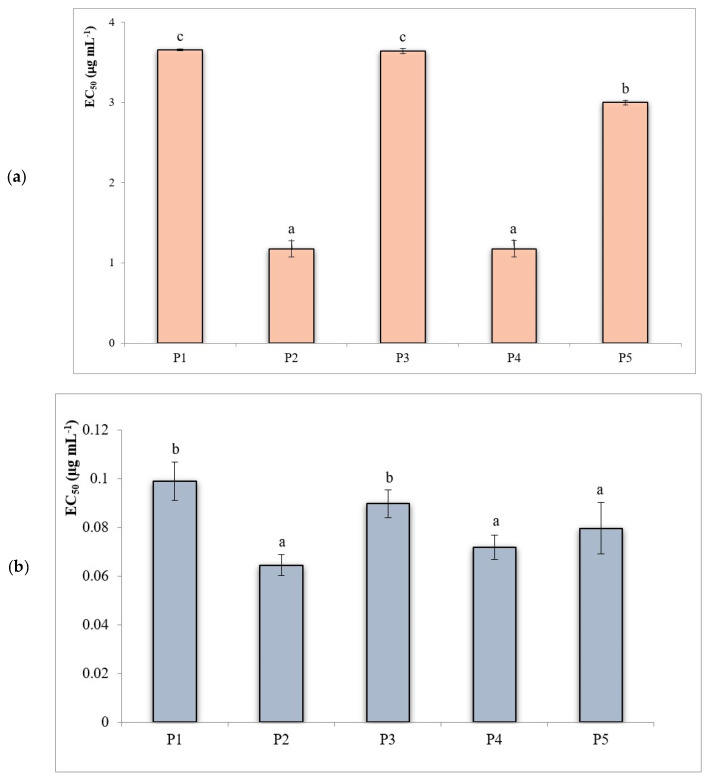
In vitro biological activities of the analyzed samples: antiradical capacity. (**a**) 2,2-diphenyl-1-picrylhydrazyl (DPPH^•^) test; (**b**), 2′-azino-bis (3-ethylbenzothiazoline-6-sulfonic acid) (ABTS^•+^) test. In both in vitro tests, antiradical capacity expressed in terms of EC_50_ (μg mL^−1^ of extract required to obtain 50% radical scavenging). In the histogram, different letters indicate significant differences (*p* < 0.05).

In order to investigate the radical scavenging properties of the different extracts analyzed, two different in vitro antiradical assays based on hydrogen and electron transfer were performed. Figure 6 shows the antiradical potential of the extracts analyzed towards synthetic chromogenic radicals DPPH^•^ and ABTS^•+^, expressed in terms of EC_50_: the lower this value, the higher the antiradical capacity of the extract. In both in vitro tests, the highest antiradical capacity values were recorded for sample P2 (EC_50_: 1.17 and 0.064 μg mL^−1^, for DPPH^•^ and ABTS^•+^, respectively), according to its highest polyphenolic content.

The antiradical potential of the extracts tested was higher than those reported by Tenuta et al. [40] for *A. unedo* berries from Southern Italy. Nonparametric correlation analysis, carried out on the entire dataset, pointed out the highest significant correlation between EC_50DPPH_ and malvidin-3-O-glucoside content of extracts analyzed (ρ: −0.886; *p* < 0.01), while no significant correlations were found among EC_50ABTS_ and the variables analyzed.

Type 2 diabetes is a form of diabetes that is characterized by high blood sugar, insulin resistance and relative lack of insulin, and it represents over 90% of diabetes cases worldwide. The reduction or inhibition of carbohydrate absorption by inhibiting digestive enzymes such as α-amylase and α-glucosidase is one of the most widely used strategies to reduce postprandial hyperglycemia [10].

**Figure 7:**
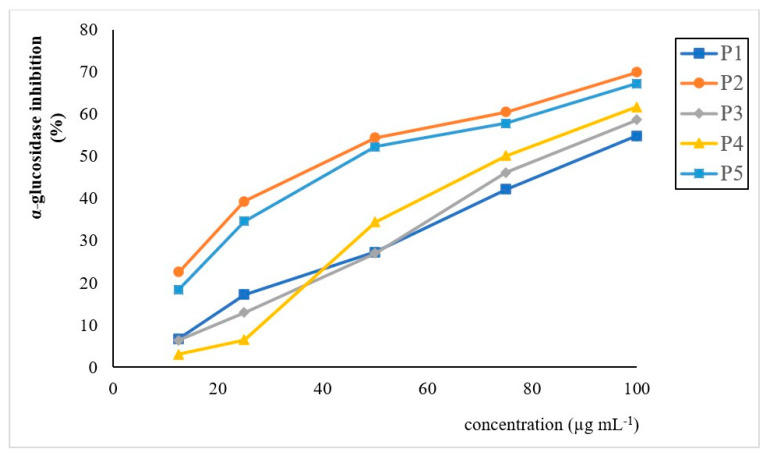
Percentage of inhibition of the a-glucosidase enzyme by extracts of *A. unedo* wild berries.

In order to explore the hypoglycemic potential of *A. unedo* wild berries, all the extracts analyzed were evaluated for their inhibitory power against α-glucosidase in the range of 0–100 mg mL^−1^. As can be seen from Figure 7, the wild accessions studied showed a peculiar inhibition trend, with P2 and P5 samples exerting the highest inhibitory power (70 and 67%, respectively).

Nonparametric correlation analysis pointed out very high significant correlations among percentage of α-glucosidase inhibition and cyanidin-3-O-rutinoside (ρ: 0.973; *p* < 0.01), TMA (ρ: 0.912; *p* < 0.01) and FLC (ρ: 0.912; *p* < 0.01), highlighting the synergistic effect of bioactive compounds present in the extracts analyzed.

### 3.5. Exploratory Data Analysis

**Figure 8:**
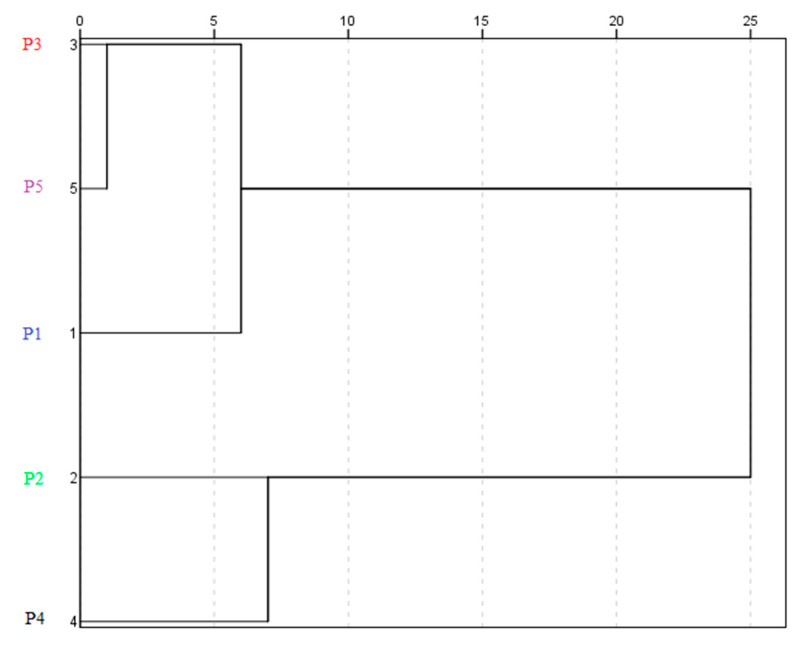
Hierarchical cluster analysis for the *A. unedo* accessions analyzed.

In the present study, HCA was initially employed to explore the organization of the analyzed samples into groups and between groups depicting a hierarchy, considering the entire normalized dataset. The resulting dendrogram is presented in Figure 8.

An overall separation was observed among the wild accessions at an average distance between 10 and 15 (two main clusters). Besides this, at an average distance ranging from 5 to 10, three clusters were observed, confirming that the nutraceutical composition and functional activities of the P1 accession were highly distinct from the remaining samples.

HCA was then followed by K-W nonparametric analysis to highlight which variables contributed most to the separation. Where significant H-values occurred, Dunn’s post hoc comparisons were conducted to determine the simple effects between clusters. Significant differences (*p* < 0.05) were observed among the three clusters for TPC, FLC, TMA, anthocyanin compounds, phenolic acids, catechin, AC_ABTS•+_ and AC_DPPH•_ (Table 2).

Based on the post hoc analysis (Table 2), it was possible to group the accessions in the study into clusters with low (Cluster 1; P1), medium (Cluster 3; P3 and P5), and high (Cluster 2; P2 and P4) nutraceutical potential.

Finally, PCA was used to visualize the hidden pattern in the experimental data and the relationships between data and samples. It was conducted on selected variables (Table 3), chosen on the basis of the analysis of the correlation and anti-image correlation matrices obtained from the standardized z-scores [18], with orthogonal rotation (varimax model).

The Kaiser–Meyer– Olkin measure verified the sampling adequacy for the analysis (KMO = 0.747; [19]). Bartlett’s test of sphericity (*p* < 0.001) showed that correlations between the considered items were sufficiently large for PCA. On the basis of eigenvalues > 1 (Kaiser’s criterion) and of the scree plot (not shown), two principal components (PCs), accounting for 93.52% of the total variance, were considered significant. Component loadings after rotation were reported in Table 3, showing the main variables differentiating the extracts.

As we can see, TPC, chlorogenic acid, catechin, malvidin-3-O-glucoside and antiradical potential (EC_50DPPH_) loaded highly on factor 1, explaining most of its variance (48.12%; rotated solution). Conversely, the remaining anthocyanins (mainly delphinidin) and TMA loaded highly on factor 2 (45.40%; rotated solution).

**Figure 9:**
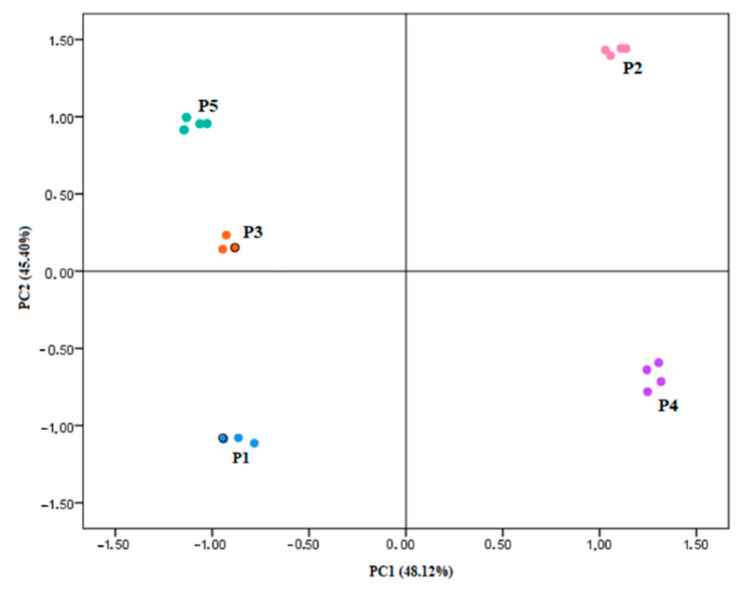
Score plot of the principal components PC1 and PC2.

Figure 9 depicts the scores for samples analyzed in a two-dimensional plot. Across PC1, a broad separation was observed among P2 and P4 samples and the remaining ones, the former having an overall higher nutraceutical profile and significant antioxidant potential. A clear sample separation was also observed across PC2, highlighting the lower content of anthocyanins of P1 and P4 accessions compared to the other ones.

## 4. Conclusions

Recently, underutilized berry species have attracted growing interest as promising sources of bioactive compounds, which, thanks to a comprehensive phytochemical evaluation, could find wider applications in various fields such as nutraceutical and cosmeceutical ones. In the present study, a phytochemical analysis coupled with unsupervised chemometric tools revealed the potential of wild berries from Italian *A. unedo* accessions as functional ingredients. The study focused on polyphenol and anthocyanin profiles as well as in vitro antiradical and hypoglycemic activities of five accessions growing in central Italy. Moreover, FTIR spectroscopy was shown to rapidly provide valuable information on biochemical composition and genetic differences among samples. Significant differences were found among samples tested for all parameters analyzed, being P2 the most promising accession from a phytochemical point of view. Furthermore, HCA and PCA allowed differentiating the accessions on the basis of their overall phytochemical profiles, to find out the variables that contribute most to this differentiation and to group the accessions according to low to high phytochemical content level.

This study aims to open further opportunities for the enhancement of the wild berries of *A. unedo*, improving knowledge about their nutraceutical properties, whose cultivation by virtue of the resilience characteristics of the species could make an important contribution to the development of local rural areas facing the imminent problems of climate change.

## Figures and Tables

**Table 1:** Phenolic composition (mg g^−1^) of *Arbutus unedo* extracts determined by high-performance liquid chromatography (mean ± DS).

Compound	P1	P2	P3	P4	P5
*Hydroxybenzoic acids (HBA)*					
Gallic acid	0.65 ± 0.01 ^b^	0.74 ± 0.01 ^b^	0.63 ± 0.01 ^b^	1.29 ± 0.02 ^c^	0.31 ± 0.03 ^a^
Syringic acid	0.09 ± 0.01 ^a^	0.14 ± 0.02 ^a^	0.11 ± 0.02 ^a^	0.26 ± 0.01 ^b^	0.47± 0.02 ^c^
Protocatechuic acid	0.35 ± 0.01 ^a^	0.45 ± 0.02 ^a^	0.31 ± 0.01 ^a^	0.59± 0.01 ^b^	0.44 ± 0.01 ^a^
*p*-Hydroxybenzoic acid	0.11 ± 0.02 ^a^	1.23 ± 0.02 ^d^	0.44 ± 0.01 ^b^	0.64 ± 0.02 ^c^	0.47 ± 0.02 ^b^
Total HBA	1.2 ± 0.1	2.56± 0.07	1.49 ± 0.05	2.78 ± 0.06	1.69 ± 0.08
*Hydroxycinnamic acids (HCA)*					
***p***-Coumaric acid	n.d	n.d	n.d	n.d	n.d
Caffeic acid	n.d	n.d	n.d	n.d	n.d
Chlorogenic acid	0.05 ± 0.01 ^b^	0.06 ± 0.01 ^c^	0.05 ± 0.02 ^a^	0.06 ± 0.01 ^d^	0.05 ± 0.01 ^a^
Ferulic acid	n.d	n.d	n.d	n.d	n.d
Total HCA	0.05 ± 0.01	0.06 ± 0.01	0.05 ± 0.02	0.06 ± 0.01	0.05 ± 0.01
*Flavan-3-ols*					
Catechin	0.82 ± 0.02 ^a^	1.72 ± 0.01 ^d^	1.04 ± 0.02 ^b^	1.34 ± 0.01 ^c^	0.87 ± 0.01 ^a^
Epicatechin	0.09 ± 0.01 ^a^	0.12 ± 0.02 ^a^	0.11 ± 0.02 ^a^	0.16 ± 0.02 ^b^	0.11 ± 0.01 ^a^
Total flavans	0.91 ± 0.03	1.84 ± 0.03	1.15 ± 0.04	1.5 ± 0.3	0.98 ± 0.02 ^b^

n.d.: not detectable. Different letters in a row indicate significant differences in the mean (*p* < 0.001).

**Table 2:** Nonparametric analysis of functional traits of *A. unedo* extracts analyzed (mean values ± standard deviation).

Variable	Cluster 1	Cluster 2	Cluster 3
TPC	18 ± 2 ^ab^	23.9 ± 0.2 ^c^	18.1 ± 0.4 ^ba^
FLC	3.0 ± 0.4 ^ab^	8.4 ± 0.2 ^ca^	7.0 ± 0.4 ^ba^
TMA	30.1 ± 0.4 ^ab^	63.1 ± 0.4 ^ba^	48.6 ± 0.1 ^c^
DEL	49 ± 1 ^abc^	88 ± 3 ^ca^	86 ± 1 ^ba^
CGC	6.3 ± 0.1 ^ac^	8.1 ± 0.3 ^ca^	7.5 ± 0.1 ^b^
CRC	9.5 ± 0.4 ^ac^	21 ± 1 ^ca^	17.5 ± 0.3 ^ba^
GA	0.60 ± 0.06 ^b^	1.00 ± 0.04 ^cba^	0.50 ± 0.01 ^a^
*p*OH-B	0.10 ± 0.01 ^abc^	0.9 ± 0.1 ^cba^	0.40 ± 0.02 ^bac^
SA	0.08 ± 0.01 ^ac^	0.20 ± 0.01 ^ca^	0.160 ± 0.008 ^b^
pCA	0.35 ± 0.01 ^ac^	0.52 ± 0.03 ^ca^	0.38 ± 0.01 ^b^
CAT	0.82 ± 0.02 ^ac^	1.53 ± 0.06 ^cba^	0.95 ± 0.01 ^bc^
CA	1.6 ± 0.2 ^abc^	1.9 ± 0.5 ^cba^	1.7 ± 0.6 ^bac^
ABTS	3.0 ± 0.2 ^cba^	2.4 ± 0.1 ^bc^	2.0 ± 0.2 ^ac^
DPPH	109.6 ± 0.3 ^ca^	35 ± 1 ^abc^	100 ± 1 ^ba^

TPC: total polyphenol content; FLC: flavan-3-ol content; TMA: total monomeric anthocyanins; *p*OH-B: *p*-hydroxybenzoic acid; CAT; catechin; CA: chlorogenic acid; DEL: delphinidin-3-O-glucoside; CGC: cyanidin-3-O-glucoside; CRC: cyanidin-3-O-rutinoside; MGC: malvidin-3-O-glucoside; DPPH: 2,2-diphenyl-1-picrylhydrazyl; GA: gallic acid; SA: syringic acid; pCA: protocatechuic acid; ABTS: 2,2’-azino-bis(3-ethylbenzothiazolin-6-sulfonic acid). Different letters in a row indicate significant differences in the mean (*p* < 0.001).

**Table 3:** Loadings of the significant measured variables on the three first principal components (PCs) *.

Variables		
	1	2
TPC	0.974	
TMA		0.825
CAT	0.862	
CA	0.848	
MGC	0.805	0.547
CRC		0.895
CGC		0.914
DEL		0.947
*p*OH-B	0.679	0.721
DPPH(EC_50_)	−0.918	
Eigenvalues	4.81	4.54
% of variance	48.12	45.40

* Rotation method: varimax with Kaiser normalization. Component loadings with absolute values less than 0.45 have been left out of the table for ease of comparison. TPC: total polyphenol content; TMA: total monomeric anthocyanins; *p*OH-B: *p*-hydroxybenzoic Acid; CAT; catechin; CA: chlorogenic acid; DEL: delphinidin-3-O-glucoside; CGC: cyanidin-3-O-glucoside; CRC: cyanidin-3-O-rutinoside; MGC: malvidin-3-O-glucoside; DPPH: 2,2-diphenyl-1-picrylhydrazyl.

## Supplementary Materials

The following are available online at https://www.mdpi.com/2223-7747/9/12/1785/s1, Table S1: Spectral features of *A. unedo* wild berries analysed.
