# Supplementary material for: Phytochemical Profile, Antiradical Capacity and α-Glucosidase Inhibitory Potential of Wild Arbutus unedo L. Fruits from Central Italy: A Chemometric Approach
Source: Plants (Basel). 2020 Dec 16;9(12):1785. doi: 10.3390/plants9121785 (PMC7766221; doi:10.3390/plants9121785)
Supplement: Supplementary file 1 [file plants-09-01785-s001.pdf]

Table S1.

Spectral features of *A. unedo* wild berries analysed.

Origin	Group Frequency wavenumber (cm^{-1})	Vibration modes
O-H	3300	Stretching vibration
C-H	2970, 2935	Symmetric and asymmetric
C-H; -CO-H	2870	Stretching vibration
C=O	1750	Stretching vibration
C-C-O	1500-1450	Stretching vibration
COO-C	1078	Stretching vibration
